# Progress of Pathogenesis in Pediatric Multifocal Atrial Tachycardia

**DOI:** 10.3389/fped.2022.922464

**Published:** 2022-06-22

**Authors:** Huaiyang Chen, Yingxu Ma, Yefeng Wang, Haiyan Luo, Zhenghui Xiao, Zhi Chen, Qiming Liu, Yunbin Xiao

**Affiliations:** ^1^Academy of Pediatrics, University of South China, Changsha, China; ^2^Hunan Children's Hospital, Changsha, China; ^3^Department of Cardiology, The Second Xiangya Hospital of Central South University, Changsha, China

**Keywords:** multifocal atrial tachycardia (MAT), inflammation, autonomic nervous, ion channel, pathogenesis, etiology

## Abstract

Multifocal atrial tachycardia (MAT) is defined as irregular P-P, R-R, and P-R intervals, isoelectric baseline between P waves, and ventricular rate over 100 beats/min. Although the prognosis of pediatric MAT in most patients is favorable, adverse outcomes of MAT have been reported, such as cardiogenic death (3%), respiratory failure (6%), or persistent arrhythmia (7%), due to delayed diagnosis and poorly controlled MAT. Previous studies demonstrated that pediatric MAT is associated with multiple enhanced automatic lesions located in the atrium or abnormal automaticity of a single lesion located in the pulmonary veins *via* multiple pathways to trigger electrical activity. Recent studies indicated that pediatric MAT is associated with the formation of a re-entry loop, abnormal automaticity, and triggering activity. The occurrence of pediatric MAT is affected by gestational disease, congenital heart disease, post-cardiac surgery, pulmonary hypertension, and infectious diseases, which promote MAT *via* inflammation, redistribution of the autonomic nervous system, and abnormal ion channels. However, the pathogenesis of MAT needs to be explored. This review is aimed to summarize and analyze the pathogenesis in pediatric MAT.

## Introduction

Multifocal atrial tachycardia (MAT) is defined as the presence of 3 or more P wave morphologies, which is characterized by irregular P-P, R-R, and P-R intervals, isoelectric baseline between the P waves, and ventricular rate over 100 beats/min on the electrocardiogram (1–4). MAT accounts for less than 1% of supraventricular tachycardia in pediatric arrhythmia, and 82% of patients diagnosed with MAT in infancy, whose lungs and heart are both immature (3, 5, 6). In previous studies, the main etiologies of MAT are gestational disease, Costello syndrome, congenital heart disease, post-cardiac surgery, cardiac anatomic abnormality, pulmonary arterial hypertension, and infectious diseases. Although the prognosis of pediatric MAT is favorable in most patients, adverse outcomes of MAT have been reported in some children, particularly the patients who are accompanied by HRAS, RYR2, RAF1, and RAS gene mutation (5, 7). The adverse outcomes of MAT include cardiogenic death (3%), respiratory failure (6%), or persistent arrhythmia (7%). Some recent studies demonstrated that pediatric MAT is associated with multiple enhanced automatic lesions located in the atrium or abnormal automaticity of a single lesion located in the pulmonary veins *via* multiple pathways to trigger electrical activity (4). However, the pathophysiology of pediatric MAT is needed to be further explored, which needs to further analyze more clinical interrelated information and conduct fundamental experiment studies (8). This review is aimed to summarize and analyze possible pathophysiological mechanisms of MAT. Therefore, this review aims to introduce the etiology of MAT at different periods of growth and the specific etiopathogenesis of MAT.

## Etiology of Mat at Different Periods of Growth

### Gestational Disease Leads to MAT

Most cases of MAT have been detected in infancy and utero, which suggests that immaturity and vulnerability of the atrial cells are responsible for the infantile-dominated distribution of MAT (4). During pregnancy, many risk factors, such as gestational hypertension and diabetes, result in fetal ischemia, hypoxia, and edema, inducing abnormal development of fetal atrial tissue, cells, and epicardial adipose tissue (9), which in turn results in fetal atrial development abnormalities and changes in signal transduction and contributes to MAT.

#### Gestational Diabetes Mellitus

Gestational diabetes mellitus (GDM) is the most common metabolic disease during pregnancy, with an overall prevalence of 6%−13% (10, 11). In patients with gestational diabetes, maternal metabolic abnormalities lead to impaired fetal cardiovascular hemodynamics through a variety of mechanisms, including decreased cardiac output and increased aortic isthmus' flow velocity and pulsation (12). Furthermore, abnormal blood glucose variability in mothers with diabetes contributes to the development of cardiac diastolic dysfunction, even when obvious cardiac hypertrophy is not observed. Further studies have shown that chronic fetal hyperinsulinemia, caused by fetal glucose elevation within gestational diabetes, increases fetal oxygen consumption, which results in fetal hypoxemia and directly prohibits the development of the cardiovascular system *via* inducing an imbalance of oxidative stress and antioxidant (13–15). Oxidative stress and antioxidation can contribute to the development of cardiovascular disease *via* injury of endothelial cells and imbalance of inflammation factors (14). Hypoxemia and ischemia can lead to atrial fibrosis and atrial remodeling *via* calcium homeostasis disorder in fibroblasts. Dysfunction of Ca^2+^ plays a key role in the pathogenesis of MAT through increasing afterdepolarization-mediated triggered activity, conduction block, and Ca^2+^-driven cardiac alternans. All these changes promote remodeling of the fetal atrium and facilitate the occurrence of atrial arrhythmia in infants (13, 16, 17).

#### Gestational Hypertension

During pregnancy, about 10% of pregnant women develop hypertension, such as chronic hypertension, gestational hypertension, and pre-eclampsia (10, 18–21). Significant diastolic dysfunction is observed in the fetus's heart in gestational hypertension due to intrauterine growth restriction and pre-eclampsia. Moreover, cardiac dysfunction is exacerbated by the deterioration of placental vascular resistance (22–25). Particularly, fetal cardiac dysfunction in pre-eclampsia is possibly due to increased afterload, which is caused by extensive vasoconstriction, resulting in an imbalance of angiogenesis, and chronic trophoblastic ischemia (22–25). Chronic trophoblastic ischemia is a key factor of fetal hypoxia and changes in the fetal signaling pathway (26), which contributes to the formation of arrhythmogenic substrates and atrial arrhythmia. Moreover, fetal hypoxemia promotes abnormal development of the cardiovascular system, which partially contributes to fatal arrhythmia in pregnancy hypertension (27).

As mentioned above, pregnancy disease, such as GDM and hypertension, impedes the development of fetal atrial tissue and epicardial adipose tissue *via* decreasing fetal nutrition and blood supply, then promote generation of arrhythmogenic substrates and disorder of signal transduction, and, finally, facilitating the occurrence of part MAT in infants of ≤ 3 months.

### Pediatric Diseases Lead to MAT

#### Costello Syndrome

Costello Syndrome (CS) is a mutation disease of HRAS with unique craniofacial features, cardiac abnormalities (65%−75%), growth retardation, dermatological, orthopedic, ocular, and neurological problems (28–31). Cardiac abnormalities usually occur in infancy and can be detected at any age, including typical hypertrophic cardiomyopathy, congenital heart defects, and arrhythmias (especially MAT or ectopic atrial tachycardia) (29). Previous studies indicated that ~9% of Costello Syndrome is complicated with MAT (3), and every MAT can result in death within the first 2 years of life (56%), but is usually self-limiting with aggressive treatment (31, 32). Another study indicated that patients being diagnosed with CS are also complicated with AT (33%) and the majority of them have MAT (64.5%) (32). The etiology of CS with MAT is dysplasia and degeneration of the conduction system, then, the abnormal conduction pathways of the atrioventricular node (32). However, the pathogenesis of CS is complex and difficult to establish animal models, and the specific pathogenesis of CS is complicated with MAT still needs further exploration.

#### Congenital Heart Disease and Cardiac Anatomic Abnormality

Previous studies indicated that ~50% of supraventricular tachycardia (SVT) are atrial tachycardia (AT), most of which occurred in children who are <6 months old (33) and are associated with structural heart disease (34). The incidence of AT in patients with congenital heart disease (CHD) is three times that in the general population, and 42% of structural heart disease is complicated with MAT (3). Furthermore, the morbidity of atrial arrhythmia is increased with aging and the complexity of CHD, both of which predict adverse outcomes of AT, such as heart failure, death, and intervention (7). Because of early diagnosis and early surgery, the prognosis of patients with CHD has greatly improved in recent decades. However, the ameliorated survival rate is accompanied by an increase in arrhythmia morbidity (35), especially MAT, which is related to a specific type of CHD, palliative surgery, and radical surgical repair (36–38). Another study demonstrated that 50% of Klippel-Feil syndrome, tetralogy of Fallot, Noonan syndrome, myocarditis, familial hypertrophic obstructive cardiomyopathy, RASopathy syndrome, and Heterotaxy syndrome have a cardiac anatomic abnormality, which facilitates the development of MAT (3, 5).

The AT is typically related to anatomical abnormalities, which are associated with the underlying cardiac defect in children with CHD (39). Firstly, the scar and fibrosis of the atrial tissue increase atrial activation, which promotes automatic disorder and the occurrence of MAT (40). Secondly, the shunt or valve reflux caused by structural abnormalities in CHD leads to ion channel dysfunction *via* inducing enlargement of the cardiac cavity, then resulting in MAT by enhanced automaticity and triggering activities (41). Thirdly, the enlargement of the cardiac cavity resulting from hemodynamic changes could increase cardiac volume, which will result in pressure overload of each cardiac cavity. The development of arrhythmogenic substrates would be induced by scar tissue, long-term volume overload and/or pressure overload, and abnormal ion channel function, which, will, finally, result in MAT (42, 43).

#### Post-cardiac Surgery

Patients with CHD surgery are prone to arrhythmia in their lifetime. Particularly, the incidence of postoperative arrhythmia is ≥48% in children who have undergone congenital heart surgery (44), among which atrial arrhythmia is more common. The risk of atrial arrhythmia in patients with CHD is ~50% before 20 years old. Many factors are increasing the risk of tachyarrhythmia after surgical treatment. For example, cardiopulmonary bypass during surgical procedures causes an acute inflammatory state and facilitates the production of pro-inflammatory factors (38). Furthermore, aortic cross forceps increase coronary artery ischemia-reperfusion injury and myocardial conduction system ischemia, both of which cause hemodynamic changes, resulting in ischemia and hypoxemia, promoting the production of arrhythmogenic substrates, and increasing the risk of MAT; over time, owing to surgical incisions, fibrosis, suture lines, baffles (45), conduits, scarring within the walls of the atria (38), and residual cardiac lesions, which induce abnormal cardiac remodeling, hypoxemia (35), and change of areas of slow conduction. All of these lead to the formation of arrhythmogenic substrates occurrence (35), which induces an increased incidence of MAT (46). MAT predominantly arises in areas of abnormal conduction around suture points, surgical incisions, or scar borders, which is commonly caused by re-entry (45, 46). The mechanism is not clear, and follow-up studies are needed to address this issue.

#### Pulmonary Arterial Hypertension

Pulmonary arterial hypertension (PAH) is a progressive and lethal disease, especially in infants, which mainly affects cardiopulmonary function, and 8%−35% of its complication is supraventricular tachycardia, particularly atrial arrhythmias being more common and severe (47, 48). PAH leads to right atrial enlargement, pulmonary vascular resistance increases, and lower cardiac index values, all of which promote the probability of occurrence of atrial arrhythmia (49–51). Recent studies indicated that legumain activated MMP2/TGF β1 signaling pathway to increase extracellular matrix (ECM) in PAH (52) and activated PI3K/Akt pathway to promote vascular remodeling in atherosclerosis (53). Another recent study has shown that legumain affects the prognosis of acute myocardial infarction by excessive ECM degradation *via* MMP2 pathway activation (54). Consequently, this review speculates that legumain plays an important role in PAH with atrial arrhythmia, but the pathogenesis is needed to be further investigated.

#### Infectious Diseases

Infants, especially at age of 3 months old or less, are susceptible to acute infectious diseases, particularly, when it is concomitant with congenital heart disease. It is more prone to immune deficiency, which increase the possibility of infectious diseases, such as respiratory system infection and digestive system infection (55). Inflammation caused by infection diseases, promoting hypercapnia, hypoxia, or adrenergic stimulation, and increases the cardiac load, which may result in right atrium enlargement and development of MAT (3, 5, 56). Chronic inflammation has been caused by sarcoidosis, systemic lupus erythematosus, and scleroderma, which could induce sympathetic nervous overactivation and accelerate myocardial cell necrosis, which triggers electrical and structural remodeling, and, finally, resulting in MAT (57).

## Etiopathogenesis of Mat

### Inflammation

**Figure 1:**
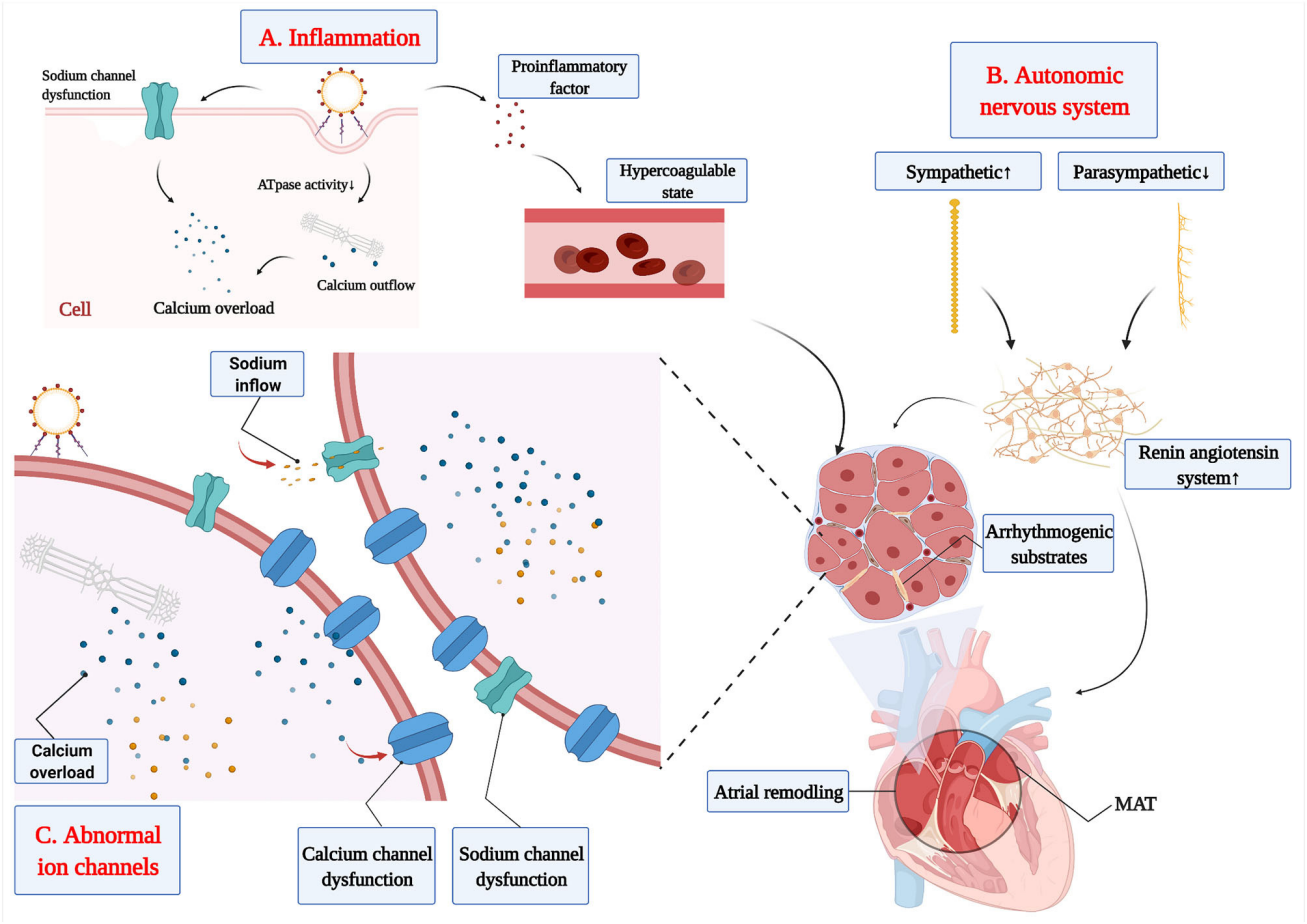
The etiopathogenesis of MAT. **(A)** The release of proinflammatory factor from inflammation increases the risk of MAT through hypercoagulable state and dysfunction of myocardial cell. **(B)** The disorder of the autonomic nervous system increases the activation of the renin-angiotensin-aldosterone system to promote the development of arrhythmogenic substrates. **(C)** The calcium channel and sodium channel dysfunction promote the development of arrhythmogenic substrates.

Inflammation increases the risk of MAT through the following mechanisms. Firstly, disorganized secretion of inflammatory cytokines promotes the release of pro-inflammatory factors, which induces disorderly physiological functions of the myocardial cell by several pathways (Figure 1A): (A) Calcium overload results in abnormal calcium handling and potentially prolonged action potential duration (58); (B) Intracellular calcium levels are increased by reduced sarcoplasmic reticulum ATPase activity; (C) Calcium is leaked from the sarcoplasmic reticulum (59); (D) Myocardial depolarization damage is caused by sodium channel dysfunction (60); (E) Reduction of heart rate variability is observed; and (F) It prolongs the interval between QTc and myocardial repolarization to disrupt the physiological functions of myocardial cells. Secondly, the proinflammatory factor is released by myocardial cell at the inflammatory response, which has a substantial influence on blood coagulation and fibrinolytic system and produces blood coagulation system activation to result in a hypercoagulable state. Part of the studies indicated that coagulation cascade is produced at the systemic inflammatory response, which is *via* a variety of ways to promote the occurrence of MAT directly or indirectly (61). Thirdly, hemodynamic changes and redistribution of the blood flow in various organs and tissues of the body are produced by an inflammatory response, which induces ischemia and hypoxia in local organs or tissues and ischemic heart diseases occurrence in severe cases. All of these facilitate the formation of arrhythmogenic substrates, which can ultimately result in enhanced automaticity, reentry, and triggered activity (62). Previous research have reported that inflammation could promote the release of inflammatory cytokines, such as TNF-α, IL-6, and IL-8, which also have been used as an important biomarker of acute infection and played a role in the prediction of AT in partial patients (62, 63). Therefore, it is necessary to further explore the role of these biomarkers in MAT and find more suitable biomarkers for predicting MAT.

### Autonomic Nervous System

The heart is innervated by the autonomic nervous system, including the sympathetic and parasympathetic nervous systems. The autonomic nervous system plays a key role in the control of heart rate and pathogenesis of multiple types of cardiac arrhythmia (64–66).

In resting state, the vagus exerts an inhibitory modulation in heart rate *via* increasing vagal output to the sinus node and limiting the influence of the sympathetic nerve, and finally making the heart rate slow and steady. The inhibitory effect of the vagus nerve is disengaged, and the vagus nerve output decreased during stress, then, resulting in increasing in heart rate (64).

Under pathological conditions, such as myocardial infarction and heart failure, neuronal remodeling and activation of the renin-angiotensin system were triggered by the decrease in cardiac output (67). Meanwhile, mechanical stretch of atrial myocardial cells occurred in heart failure (60, 68), which could stimulate the synthesis of nerve growth factors to promote sympathetic germination and over-innervation (69). Sympathetic over-innervation enhances Ca^2+^ transient and early after depolarization, which could shorten the atrial effective refractory period and increase the effective refractory period dispersion (66, 70, 71). Finally, all of these can promote the production of arrhythmogenic substrates, which is an important factor in the development of MAT. Hypertension is characterized by peripheral vascular resistance increases, sympathetic activation enhancement, and vagus activation reduction, which increases susceptibility to atrial arrhythmia (72). Furthermore, excessive sympathetic enhancement increases the activation of the renin-angiotensin-aldosterone system, which will induce atrial electrical and structural remodeling (73), and, finally, promote the production of arrhythmogenic substrates in MAT (Figure 1B).

### Abnormal Ion Channels

The ion channels are critical to maintaining cell excitability and the propagation of electrical impulses spread in cardiac excitable cells (74). Under pathological conditions, ion channel expression and function could be altered by pathogenesis, such as inflammation, electrolyte disturbance, and autonomic nervous system imbalance (71), which then result in the development of MAT.

Although the relationship between abnormal ion channels and MAT has not fully been elucidated, there has been achieved significant progress in the functional involvement of ion channels in MAT. Voltage-gated sodium channels determine the magnitude and slope of action potential rise, which is particularly important in controlling conduction velocity and maintaining appropriate excitability in the myocardium (74). Atrial ischemia and hypoxia enhance the excitability of atrial myocytes and promote the slow inflow of a small number of sodium ions to these cells, which decrease resting membrane potential and enhance the automaticity of fast-responding cells, finally resulting in MAT. Electrolyte disturbances caused by various causes, such as hypokalemia and acidosis, would increase intracellular potassium ion outflows. However, the patients with severe hypokalemia suffer from severe potassium deficiency, both inside and outside the atrial cell. Then, cells inhibit potassium ion outflow to ensure physiological metabolism, which will decrease atrial resting membrane potential, slowing conduction velocity and prolonging repolarization (75, 76). Then, the threshold of myocardial cell excitation decreases with lower atrial resting membrane potential, which will induce AP more easily. Ectopic electrical activity will be a triggered in multiple cardiac myocytes based on existing atrial remodeling, which will trigger multiple pathways to promote MAT. Furthermore, the dysfunction of Ca^2+^ plays a key role in the pathogenesis of atrial arrhythmia (77) through increasing after depolarization-mediated triggered activity, conduction block, and Ca^2+^-driven cardiac alternans (78) (Figure 1C). Severe hypokalemia or myocardial injury inhibit the influx of calcium in slow response cells of the sinoatrial node, which relates to enhancement of atrial ectopic excitability and formation of reentry loop, finally resulting in MAT. Inflammation reduces sarcoplasmic reticulum ATPase activity, thereby increasing intracellular calcium levels, which induces calcium leakage from the sarcoplasmic reticulum (59). Moreover, the abnormal handling of calcium generates calcium overload and I_CaL_ downregulation, which can result in MAT *via* modulating atrial action potential repolarization, action potential duration, and automaticity (58).

## Conclusion

From fetal to infants <3 months old, the development of atrial tissue has not been completed, and the atria are immature and vulnerable period. At this stage, the development of atrial is extremely vulnerable, a variety of diseases could promote the development of arrhythmogenic substrates *via* various causes to the disorder of hemodynamics, ischemia hypoxia for tissues, abnormal cell metabolism, autonomic nervous system dysfunction, and abnormal ion channels, which will result in the formation of reentry loop, abnormal automaticity, and triggering activity. However, fetal atrial tissue has a strong ability to regenerate and differentiate and MAT is self-limited, so most patients with MAT have the favorable outcome. This review makes clinicians more careful about the diagnosis of MAT in pediatric patients, who had the gestational disease, congenital heart disease, cardiac anatomic abnormality, post-cardiac surgery, pulmonary hypertension, or infectious diseases, and give clinical intervention as soon as possible to avoid the occurrence of cardiogenic death, respiratory failure, and persistent arrhythmia. However, the pathogenesis of pediatric MAT, particularly in ion channel, still needs further studies, and explore the pathway to MAT prevention and better therapeutic target.

## Author Contributions

HC, YM, and YX contributed to the study. HC wrote the first draft of the manuscript. YM wrote sections of the manuscript. All authors contributed to manuscript revision, read, and approved the submitted version.

## Funding

This study was funded by Hunan Clinical Research Center for Children's Cardiovascular Diseases (2021SK4019), Major projects of Hunan Province (2020SK1013, QL), the Hunan Provincial Health Commission Project (No. 20200483), Project of Hunan Provincial Research on Chinese Medicine (No. 201914), and 2019 National Medical Service, and Support Capacity Improvement Project: Children's Difficult Diagnosis and Treatment Center.

## Conflict of Interest

The authors declare that the research was conducted in the absence of any commercial or financial relationships that could be construed as a potential conflict of interest.

## Publisher's Note

All claims expressed in this article are solely those of the authors and do not necessarily represent those of their affiliated organizations, or those of the publisher, the editors and the reviewers. Any product that may be evaluated in this article, or claim that may be made by its manufacturer, is not guaranteed or endorsed by the publisher.
